# A peculiar case of onychomycosis caused by *Cladosporium halotolerans*

**DOI:** 10.31744/einstein_journal/2023RC0326

**Published:** 2023-11-10

**Authors:** Gilglécia Novaes Pereira Santana, Catarina Glauce Martins Neff, Valeria Petri, Fernanda Aparecida Vieira Fernandes, Olga Fischman Gompertz, Marina de Moura Bello, Ruan Campos Monteiro, Daniel Archimedes Da Matta, Marília Marufuji Ogawa, Luis Henrique Barbizan de Moura, Mário Roberto de Sousa Trindade, Domingos Jordão, Zoilo Pires de Camargo

**Affiliations:** 1 Universidade Federal de São Paulo São Paulo SP Brazil Universidade Federal de São Paulo , São Paulo , SP , Brazil .; 2 Hospital Israelita Albert Einstein São Paulo SP Brazil Hospital Israelita Albert Einstein , São Paulo , SP , Brazil .; 3 Hospital de Heliópolis São Paulo SP Brazil Hospital de Heliópolis , São Paulo , SP , Brazil .

**Keywords:** Onychomycosis, Cladosporium, Cladosporium halotolerans, Cladosporium sphaerospermum, Nail diseases, Phenotype, Spectrometry, mass, matrix-assisted laser desorption-ionization

## Abstract

A 49-year-old patient with changes in the nails of the hallux for 10 years was diagnosed with onychomycosis. The identity of the causative agent was confirmed as *Cladosporium halotolerans* from the *Cladosporium sphaerospermum* species complex using molecular techniques. MALDI-TOF identified the agent as *C. sphaerospermum* complex species. Overall, species such as onychomycosis agents should attract special attention to avoid mistakes in the identification process while considering a probable contaminant as responsible for the disease. These species deserve attention since there are rare descriptions of them as causes of onychomycosis. It is important to recognize them as causes of disease and not just as a probable contaminant.

## INTRODUCTION

Onychomycosis is a common infection and accounts for most of nail diseases. It is usually caused by dermatophytes and yeasts, although it is also associated with non-dermatophyte filamentous fungi (NDFF). Recently, the incidence of onychomycosis caused by NDFF has increased, which is responsible for 2–12% of all nail fungal infections. ^( 1 )^

*Cladosporium* sp., a dematiaceous fungus, is one of the most cosmopolitan taxa and is reported to be abundant in the atmosphere. ^( 2 )^ Its dark color due to the melanic pigment in its cell wall is considered a virulence factor. ^( 3 )^ *C. halotolerans* has rarely been described as a disease agent. Melanin is recognized as a virulence factor. It ranges from its ability to promote diseases and its ability to prolong its life and adapt to several regions, making it stable and resistant. Some strains can occasionally cause cutaneous and cerebral phaeohyphomycosis regardless of the immune status of the host. ^( 4 )^ Nevertheless, onychomycosis caused by dematiaceous fungi has rarely been described in the literature. ^( 5 )^

## CASE REPORT

**Figure 1:**
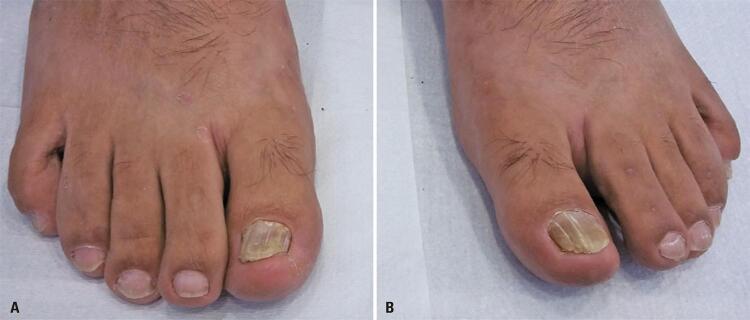
A and B) Distal lateral subungual onychomycosis of the right and left hallux

A 49-year-old male patient, retired, with psoriasis vulgaris for 15 years, and was being treated with cyclosporine 200mg/day complained of nail changes in both hallux for 10 years. He was previously diagnosed with onychomycosis and underwent irregular treatment with oral antifungal drugs without improvement. Clinical dermatological examination showed chromonychia, onycholysis, and thickening of the nail plates of both hallux, characteristic of distal lateral subungual onychomycosis ( Figure 1 ). No other alterations led to suspicions of nail psoriasis.

**Figure 2:**
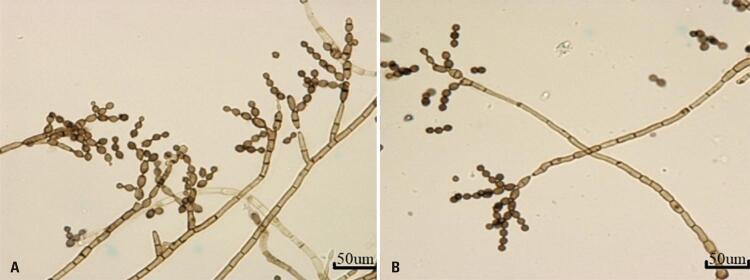
A and B) *Cladosporium* spp microculture showing the same morphologies: dark conidiophores with branches at the apex and dark, ovoid, or cylindrical branched conidiophores

A microculture was performed to identify the morphological characteristics of the agent. Direct microscopic examination (DME) of culture slides with potassium hydroxide (KOH) without staining revealed dark conidiophores with branches at the apex; and dark, branched, ovoid or cylindrical conidiospores ( Figure 2 ), which are phenotypic characteristics of *Cladosporium* spp. ^( 6 )^

Nail scrapings were collected three times at one-month interval to confirm the identity of the causative agent. DME revealed the presence of dematiaceous septate hyphae. When cultivated on Sabouraud dextrose agar (SDA) with chloramphenicol and potato agar at 27 ^o^ and 37 ^o^ C, colony surface was olive brown and olive black reverse with velvety appearance.

Morphologically similar species were distinguished using molecular techniques and MALDI-TOF mass spectrometry, which initially identified the agent as *Cladosporium endophytica* and *Cladosporium sphaerospermum* , respectively. Re-identification was performed via sequencing of the ITS1 (5′–TCCGTAGGTGAACCTGCGG–3′) and ITS4 (5′–TCCTCCGCTTATTGATATGC–3′) regions of the rDNA. ^( 7 )^ The obtained sequence was then subjected to a BLAST search (available at https://blast.ncbi.nlm.nih.gov/) and compared to those available in GenBank (NCBI, USA). Phylogenetic analysis showed that the agent had 99% similarity with *C. halotolerans* belonging to the *Sphaerospermum* complex, confirming the identity of the agent. The sequence was deposited to GenBank (accession number: MT378273). Meanwhile, identification via MALDI-TOF MS was performed using the Biotyper 3.4 software (Multiflex Bruker Daltonics GmbH) and Online MSI Platform (IHEM).Protein extraction was performed according to the MALDI-TOF MS protocol for online identification by the Marseille Teaching Hospital, France. ^( 7 )^

A new appointment was scheduled after confirmation of the etiologic agent. Initial treatment with oral itraconazole 200mg/day for 90 days was prescribed. However, follow-up was not possible because the patient did not return for future appointments.

This case study was approved by the Ethics Committee of *Universidade Federal de São Paulo* (CAAE: 04089218.5.0000.5505; # 3.116.259).

## DISCUSSION

*C. halotolerans* was previously described by Penzig ^( 8 )^ from the leaves and branches of a decomposing citrus in Italy. A few reports unequivocally prove that *C. halotolerans is* a human pathogen. ^( 9 )^

Zalar et al. ^( 9 )^ suggested that the substrates of *C. halotolerans* , which can colonize any available substrate, are distributed in the air, although this organism may have a natural niche elsewhere. Onychomycosis caused by dematiaceous fungi is rare. For laboratory diagnosis, whether the fungus is the true etiologic agent of onychomycosis should be confirmed by repeating the tests using a new sample. ^( 5 )^ *C. halotolerans* , a species of medical interest, is associated with skin infections such as phaeohyphomycosis; however, it is a rare causative agent of onychomycosis. ^( 4 )^ Sandoval-Denis et al. ^( 10 )^ analyzed 92 clinical isolates of superficial tissue from the United States using phenotypic and genotypic characterization and reported that *C. halotolerans* (15%) was the most frequent isolated species. The most frequent anatomical site of isolation was the respiratory tract (55%), followed by superficial (28%) and deep (15%) fluids.

*C. halotolerans* and *C. sphaerospermum* have always been considered as contaminants and not etiologic agents of onychomycosis. A previous study established the diagnostic criteria for nail infection, including microscopic identification using DME, isolation and cultivation of the fungal agent from three consecutive samples, and confirmation using molecular techniques. ^( 10 )^ The present case had unusual characteristics, indicating that an unusual fungus had caused the disease occurring in an uncommon location, and in a patient who did not report. The agent grew in SDA and potato agar at 27–37°C, contrary to the results of Sandoval-Denis et al., ^( 10 )^ wherein no growth was observed at a temperature below 32°C and above 35°C for this species complex.

Phylogenetic reconstruction of the isolates identified *C. halotolerans* as a member of the *Sphaerospermum* species complex. The ITS regions present interesting characteristics for identifying fungi at the molecular level. Through this approach, a diagnosis is established, the species is differentiated, and the clade to which it belongs is identified. In contrast, identification via MALDI-TOF MS was only possible on the MSI-IHEM online platform, where *C. sphaerospermum* was identified.

Few studies have investigated the susceptibility of these species. Sandoval-Denis et al. ^( 10 )^ evaluated the antifungal susceptibility of *C. sphaerospermum* species complex and found that itraconazole was effective in treating infections. Based on our results, we highlight that no established susceptibility patterns are available for this species, as well as for dermatophytes and NDFF. Owing to the increasing number of immunocompromised patients, many fungal species that are originally considered contaminants are now considered mycotic agents and can also affect immunocompetent patients. ^( 10 )^

## CONCLUSION

The presence of *C. halotolerans* in a case of onychomycosis requires attention in all patient groups. Clinical and laboratory correlations, successive sample collection following the recommended intervals, and isolation of the fungus without any other agent involved in successive fungal cultures are important. Therefore, a probable contaminant may also be responsible for onychomycosis. Our results contribute to our understanding of this agent, which has rarely been reported as a human pathogen.
